# Bosutinib-Induced Stevens-Johnson Syndrome and Evidence of Tolerance to a Structurally Dissimilar Tyrosine Kinase Inhibitor

**DOI:** 10.7759/cureus.23288

**Published:** 2022-03-18

**Authors:** Karol Avila-Castano, Dan Morgenstern-Kaplan, Ismael Carrillo-Martin, Alexei Gonzalez-Estrada

**Affiliations:** 1 Division of Pulmonary, Allergy, and Sleep Medicine, Department of Medicine, Mayo Clinic, Jacksonville, USA

**Keywords:** skin reactions, tyrosine kinase inhibitors, chronic myeloid leukemia, scar, stevens-johnsons

## Abstract

Bosutinib is a breakpoint cluster region-Abelson gene (BCR-ABL) tyrosine kinase inhibitor (TKI) used for the treatment of chronic myeloid leukemia (CML). Patients on TKIs may develop severe cutaneous adverse reactions (SCARs). A 73-year-old female with CML treated with a second-generation TKI (bosutinib) was evaluated after developing fever and a maculopapular exanthema with skin-peeling affecting her lips, oral mucosa, and genitals 10 days after starting the medication. She required hospitalization, bosutinib discontinuation, and management with intravenous corticosteroids and antibiotics. Patch testing was contraindicated due to the severity of the reaction. The patient was subsequently challenged with first-generation TKI along with careful observation without any adverse reactions. She has not reported any adverse reactions while on therapy in the last two years. In patients who have suffered from SCARs, the suspected triggers must be avoided in all instances. In some cases, the underlying condition limits the use of alternative agents, but low-concentration patch testing may help guide alternative therapies within the same medication group. There appears to be a low cross-reactivity among generational TKIs, and our patient benefited from treatment with a structurally dissimilar alternative TKI for her CML.

## Introduction

Bosutinib, a second-generation oral tyrosine kinase inhibitor (TKI) that targets SRC/Abelson (ABL) kinases, is used as a first-line treatment for chronic myeloid leukemia (CML) [1]. Treatment with TKIs may cause severe cutaneous adverse reactions (SCARs), such as Stevens-Johnson syndrome (SJS) or toxic epidermal necrolysis (TEN). We present a case of bosutinib-induced SJS in a patient who tolerated a structurally dissimilar TKI.

## Case presentation

**Figure 1:**
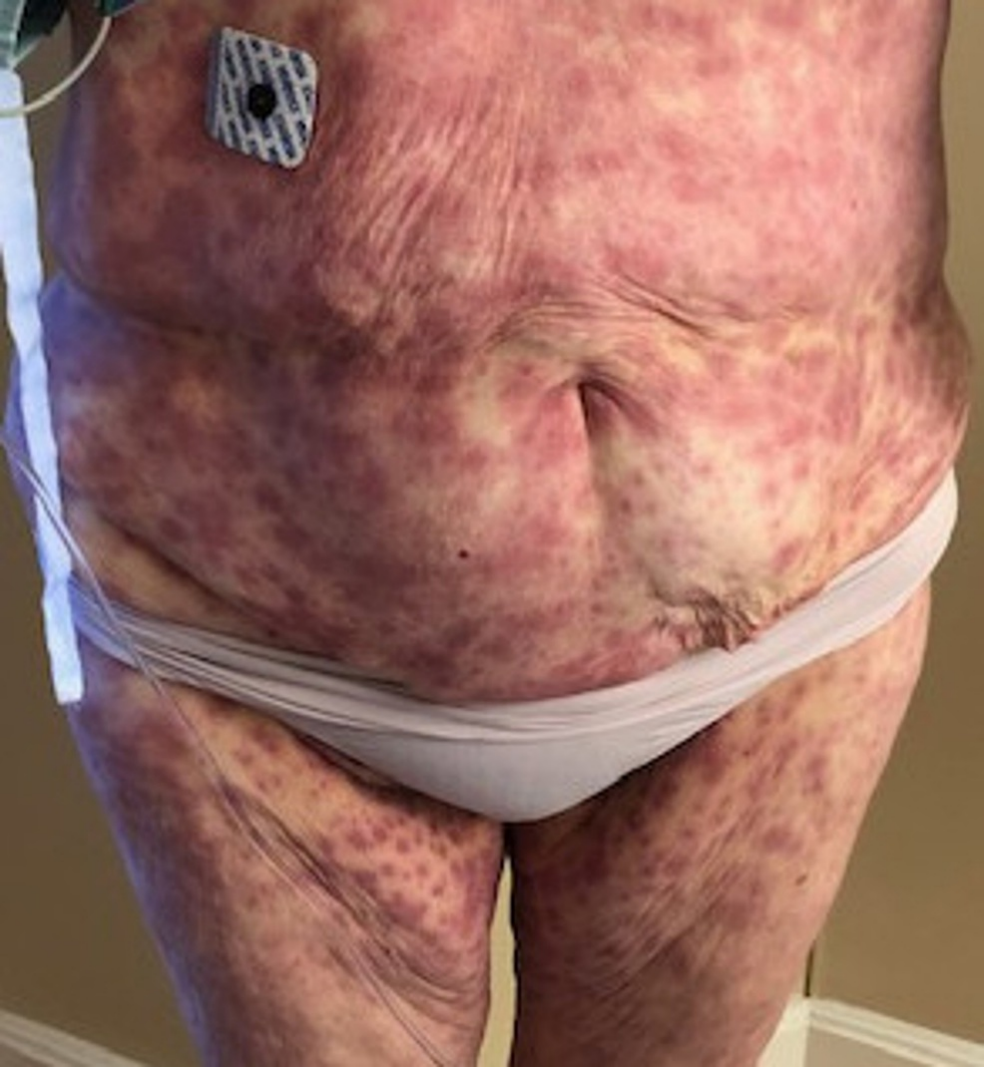
Photograph of the abdomen and lower extremity Clinical photograph showing the erythematous macules in the abdominal region, characteristic of Stevens-Johnson syndrome. The dermatologic lesions were present on most of the body and improved upon discontinuation of bosutinib and inpatient treatment

A 73-year-old female with CML treated with bosutinib, non-insulin-dependent diabetes, hyperlipidemia on fibrates, atrial fibrillation on carvedilol, apixaban, and digoxin presented to an outside hospital with a rash and fever 10 days after starting bosutinib 400 mg orally. No new medications had been started in the period spanning three months prior to the initiation of bosutinib. Physical examination showed erythematous macules with mucosal erosions in lips and genitals; Nikolsky's sign was positive (Figure 1). Based on the clinical presentation and physical exam, a diagnosis of SJS secondary to bosutinib was made. Bosutinib was discontinued and the patient was treated with methylprednisolone 125 mg IV, piperacillin/tazobactam 3.375 g IV once, diphenhydramine 25 mg IV, famotidine 20 mg IV, and vancomycin 1500 mg IV. She was discharged after five days and the rash improved within nine days.

The patient was deemed not a candidate for a bone marrow transplant and she declined low-dose TKI. After almost three months, the patient was evaluated at our allergy clinic.

The patient was invited to participate in the medical decision-making. She decided to undergo an open long challenge with imatinib, a first-generation TKI, for 10 days with close observation. After that, she did not develop any adverse reactions. The patient has tolerated therapy with imatinib 400 mg daily, without further adverse reactions for the past two years.

## Discussion

The most commonly reported adverse reactions of bosutinib include gastrointestinal symptoms (abdominal pain, diarrhea, nausea, and vomiting), thrombocytopenia, anemia, and hepatic failure [2]. Cutaneous adverse reactions secondary to bosutinib include non-specific manifestations such as edema or rash that occur in approximately 22% of the patients [2,3].

SJS/TEN are cutaneous reactions caused by a type IV subtype C hypersensitivity reaction with cytotoxic T cells acting as the major mediator of keratinocyte apoptosis [4,5]. Imatinib is a common first-line treatment for CML that has been associated with SCARs such as SJS/TEN [6]. Delayed skin testing, patch testing, and drug provocation test on patients diagnosed with SCARs are not routinely recommended due to a high risk of mortality [7]. However, a low concentration of patch testing after SJS/TEN may be considered in special situations if there is a potential diagnostic benefit from the information to be obtained [8]. In patients with delayed cutaneous hypersensitivity drug reactions to imatinib and dasatinib, patch tests at 1%, 5%, and 10% have been used in previous studies [9]. Small inhibitor molecule therapy with agents similar to TKIs can cause a vast array of dermatologic toxicities, ranging from simple maculopapular rashes to severe reactions such as SCARs [10].

In-vitro studies, such as enzyme-linked immunosorbent spot assay and flow cytometry, conducted on peripheral blood mononuclear cells may help in identifying the causative agent in the setting of SJS [7,11]. These tests measure cytokines (FasL, granulysin, IL-15, TNF-α), which are involved in the pathophysiology of SJS/TEN and have the potential to identify causal agents in vitro [5,7,11].

## Conclusions

Patients treated with TKIs can develop SCARs such as SJS. Utilizing a structurally dissimilar TKI may be an option when no equally efficacious alternatives exist after its risks and benefits have been discussed with the patient.
